# Generalizable transfer learning of automated tumor segmentation from cervical cancers toward a universal model for uterine malignancies in diffusion-weighted MRI

**DOI:** 10.1186/s13244-022-01356-8

**Published:** 2023-01-24

**Authors:** Yu-Chun Lin, Yenpo Lin, Yen-Ling Huang, Chih-Yi Ho, Hsin-Ju Chiang, Hsin-Ying Lu, Chun-Chieh Wang, Jiun-Jie Wang, Shu-Hang Ng, Chyong-Huey Lai, Gigin Lin

**Affiliations:** 1 Department of Medical Imaging and Intervention, Chang Gung Memorial Hospital at Linkou and Keelung, 5 Fuhsing St., Guishan, Taoyuan, 33382 Taiwan; 2 Department of Medical Imaging and Radiological Sciences, Chang Gung University, Taoyuan, 33302 Taiwan; 3 Clinical Metabolomics Core Laboratory, Chang Gung Memorial Hospital at Linkou, 5 Fuhsing St., Guishan, Taoyuan, 33382 Taiwan; 4 Gynecologic Cancer Research Center, Department of Obstetrics and Gynecology, Chang Gung Memorial Hospital at Linkou and Chang Gung University, 5 Fuhsing St., Guishan, Taoyuan, 33382 Taiwan; 5 Department of Radiation Oncology, Chang Gung Memorial Hospital at Linkou and Chang Gung University, 5 Fuhsing St., Guishan, Taoyuan, 33382 Taiwan

**Keywords:** Magnetic resonance imaging, Deep learning, Uterine neoplasms

## Abstract

**Purpose:**

To investigate the generalizability of transfer learning (TL) of automated tumor segmentation from cervical cancers toward a universal model for cervical and uterine malignancies in diffusion-weighted magnetic resonance imaging (DWI).

**Methods:**

In this retrospective multicenter study, we analyzed pelvic DWI data from 169 and 320 patients with cervical and uterine malignancies and divided them into the training (144 and 256) and testing (25 and 64) datasets, respectively. A pretrained model was established using DeepLab V3 + from the cervical cancer dataset, followed by TL experiments adjusting the training data sizes and fine-tuning layers. The model performance was evaluated using the dice similarity coefficient (DSC).

**Results:**

In predicting tumor segmentation for all cervical and uterine malignancies, TL models improved the DSCs from the pretrained cervical model (DSC 0.43) when adding 5, 13, 26, and 51 uterine cases for training (DSC improved from 0.57, 0.62, 0.68, 0.70, *p* < 0.001). Following the crossover at adding 128 cases (DSC 0.71), the model trained by combining data from adding all the 256 patients exhibited the highest DSCs for the combined cervical and uterine datasets (DSC 0.81) and cervical only dataset (DSC 0.91).

**Conclusions:**

TL may improve the generalizability of automated tumor segmentation of DWI from a specific cancer type toward multiple types of uterine malignancies especially in limited case numbers.

**Supplementary Information:**

The online version contains supplementary material available at 10.1186/s13244-022-01356-8.

## Key points

1. Transfer learning (TL) improves performance of tumor segmentation on diffusion-weighted imaging (DWI) especially in limited case numbers.
2. Training a model by combining sufficient data of different cancers exhibited the highest performance for segmenting mixed cervical and uterine datasets and also improved the pretrained cervical model.
3. The TL model with fine-tuning the early layers of the encoder part outperformed those by fine-tuning the other layers.

**Supplementary Information:**

The online version contains supplementary material available at 10.1186/s13244-022-01356-8.

## Introduction

Magnetic resonance imaging (MRI) plays crucial roles for gynecologic malignancies, in the initial or preoperative staging, response monitoring and surveillance for recurrence [1], by providing anatomical details and functional parameters [2]. MRI-derived target volumes are increasingly used for radiation treatment planning for cervical cancer, based on the accurate tumor contouring and precisely evaluating tumor extension [3]. In addition to tumor localization, MRI-based radiomics has potential to differentiate benign versus malignant uterine tumors [4] and provides useful information for the outcome prediction [5]. However, the radiomics pipeline involves extracting features from large number of images [6], related to potential discrepancies of manual contouring among human readers [7], highlighting the pressing need for a fully automatic method for tumor segmentation.

Convolutional neural networks (CNNs) have shown promise as alternatives for image segmentation [8, 9]. However, building a CNN model requires far more annotated datasets than are available in medical imaging [10]. Furthermore, training a segmentation model in a three-dimensional volume dataset, such as CT or MRI, requires manual labeling in a slice-by-slice manner, which is extremely labor intensive. Therefore, reducing the size of the training dataset is necessary to boost training efficiency. Transfer learning (TL) is an approach to address this problem, which uses features learned from a source domain as a pretrained model and transfers them to another domain [11]. Several studies have shown that TL has potential to overcome the requirement of large data in medical imaging [12–14]. To the best of our knowledge, no study has investigated the usefulness of TL in studying different cancers. Because the majority of tumors exhibit high intensity on diffusion-weighted (DW) imaging and low intensity on the apparent diffusion coefficient (ADC) map, we assume that various tumors have common features in certain layers of the network, and knowledge regarding one tumor type can be transferred for the study of another type. Previous study has established a model of tumor segmentation in cervical cancer (CX) with DW images [15]. In the current study, we hypothesized that the pretrained model of CX dataset can be transferred to all cervical and uterine malignancies, with a reduced sample size of labeled images.

This study investigated the generalizability of transfer learning (TL) of automated tumor segmentation from cervical cancers toward a universal model for all cervical and uterine malignancies in diffusion diffusion-weighted magnetic resonance imaging (DWI). Specifically, we investigated whether TL can reduce the data size required for the target domain, and we deciphered which parts of CNN layers should be fine-tuned to achieve adequately accurate tumor segmentation. Finally, the model performance of TL was compared with an aggregated model, which was trained using the combined cervical and uterine datasets, for the universal tumor segmentation of malignant uterine tumors on MRI.

## Materials and methods

### Patients

This exploratory study retrospectively analyzed the dataset of patients with cervical or uterine malignancies at a tertiary referral center between July 2010 and January 2018. The Institutional Review Board approved the study, and informed consent was waved. The experiments were performed using two cohorts of patients: (1) cervical dataset, which was used as the source domain for the pretrained model. This dataset was used for establishing the tumor segmentation model in previous report [15]; the dataset included the data of 144 patients with cervical cancer for training and the data of 25 patients for testing; (2) uterine dataset, the target domain for transfer learning experiments.

The inclusion criteria for patients in the uterine dataset were as follows: (a) female sex, (b) age of 20–80 years, and (c) clinical diagnosis of uterine malignancies. The exclusion criteria were as follows: (a) contraindicated for an MRI study due to a cardiac pacemaker or cochlear implantation; (b) post major pelvic surgery, total hip replacement, or magnetic substance implantation in the pelvis; (c) significant major systemic disease, such as renal failure, heart failure, stroke, acute myocardial infarction/unstable angina, poor controlled diabetes mellitus, and poor controlled hypertension; and (d) pregnant or breast-feeding.

Of 345 consecutive patients enrolled, we excluded 16 patients who had no visible tumors and nine patients susceptible to artifacts in DW imaging. Thus, the data of 320 patients in the uterine dataset were included in the final analysis (Fig. 2). Among them, 256 patients (80%) were randomized to the training dataset, and the remaining 64 patients (20%) were included in the testing dataset. All data were exported anonymously.

### MRI data and image annotation

MRI studies were performed using two MRI scanners: Skyra (*n* = 248) and Trio TIM (*n* = 72) (Siemens Healthineers). All patients underwent the standard MR protocol from Chang Gung Memorial Hospitals following the guide of European Society of Urogenital Radiology for female pelvis imaging [16]. The imaging protocol included T1-weighted, T2-weighted, DW images and contrast-enhanced T1-weighted images acquired in sagittal and axial planes. The DW imaging utilized single-shot echo planar imaging with *b*-values = 0 and 1000 s/mm^2^ to generate ADC map. (repetition time/echo time, 3700–8200 ms/65–85 ms; slice thickness/interval, 4 mm /1 mm; field of view, 200 × 200 mm; matrix, 256 × 256). The slice sections ranged 14–22 to cover the whole tumor for each patient. The sagittal DW images and the corresponded ADC maps of each slice were used as input sources for training and testing.

Regions of interest (ROIs) of tumor contours were delineated by the consensus of two gynecologic radiologist (Y.L.H. and G.L. with 7 and 13 years of experience in gynecology, respectively) using an in-house developed interface in MATLAB (Mathworks, Natick). Both readers were blinded to clinical outcomes. We avoided the ROIs contaminating the adjacent normal endometrium and myometrium and excluded the normal cervical stroma when studying the cervical invasion. The labeled ROIs were used as the ground truth for the model training.

### Network and training

In an optimization study, we explored the performance of U-Net and DeepLab V3 + architectures for tumor segmentation in cervical cancer. Finally, the DeepLab V3 + architecture was adopted because it produced higher preliminary accuracies (Additional file 1). The DW MRI with *b*-values of 0 and 1000 s/mm^2^ and ADC images were used as three-channel input sources for training. Xception was used as the backbone (first 356 layers) of the DeepLab V3 + network. The networks were trained with weight randomization and stochastic gradient descent Adam Optimizer method [17]. The signal intensities of all images were normalized to a mean = 0 and standard deviation = 1 [18]. We implanted data augmentation on each training image set, such that six times of image data were generated (20°, − 20°, 60°, − 60°, and horizontal flip). Finally, 10,164 images from the 256 patients in the uterine training dataset were used for training. The learning rate was 0.001, and the number of epochs until convergence was 100, with batch sizes of 2. The network was trained using Keras 2.1.4 written in Python 3.5.4 and TensorFlow 1.5.0. The code for the DeepLab V3 + model is available at https://github.com/bonlime/keras-deeplab-v3-plus.

### Model experiments

The pretrained model was established using DeepLab V3 + for cervical dataset (n = 144). We performed three combinations of model training and prediction: (a) UT-only model: training from scratch using the uterine dataset without TL from cervical model; (b) TL model: using the pretrained cervical model and fine-tuning of certain layers using the uterine dataset; and (c) Aggregated model: training from scratch by using the combined cervical and uterine datasets. This model was proposed to test the generalization for both cervix and uterine cancers.

**Fig. 1:**
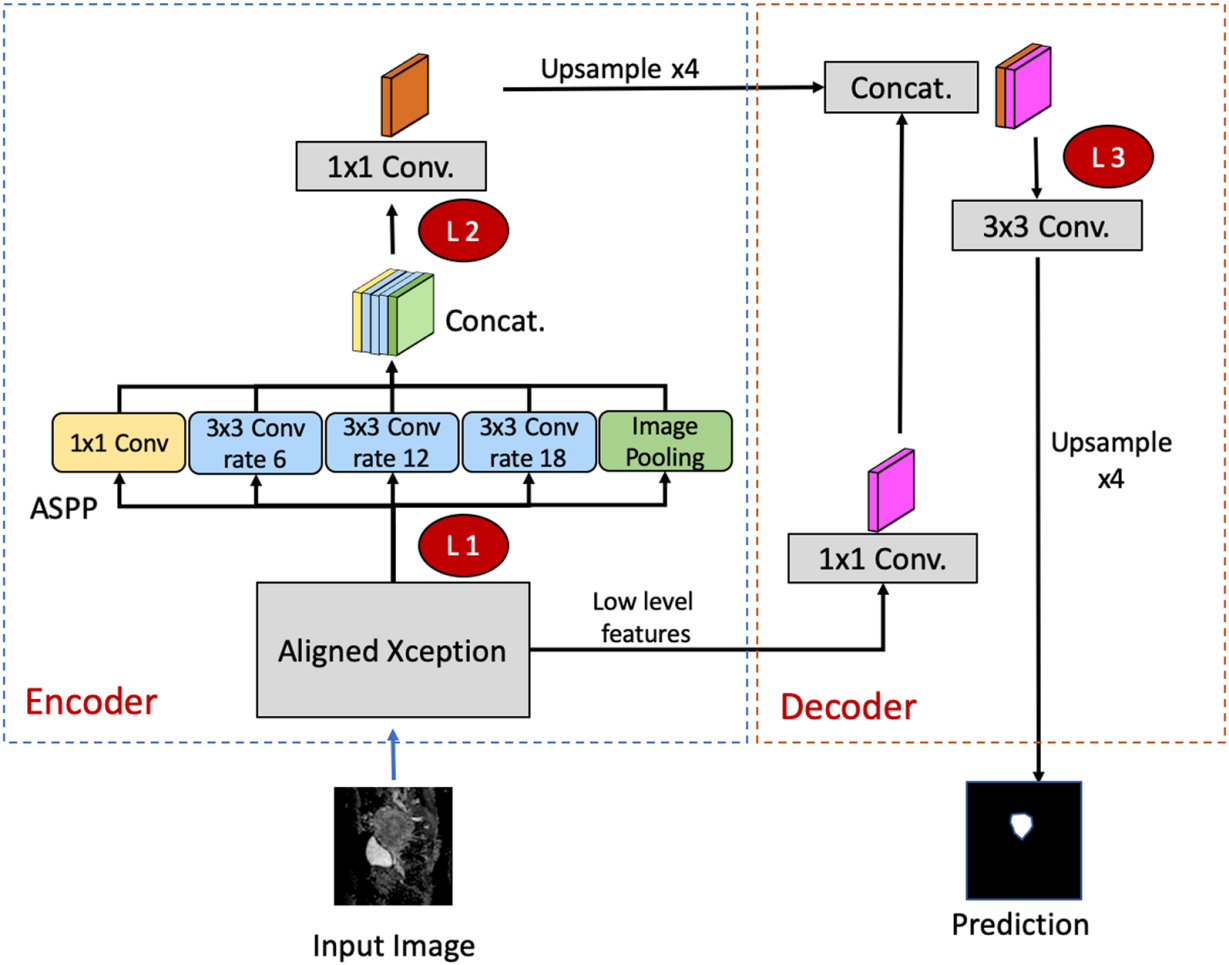
The DeepLab V3 + network architecture in the experiment. The red circle with annotations of L1, L2, and L3 denotes the cutoff levels in which the previous layers are frozen, whereas the following layers are fine-tuned based on the target dataset. L1: an early layer in the encoder following the Xception model. L2: a deep layer following the ASPP at the end of the decoder. L3: the layer at decoder initiation. ASPP: Atrous Spatial Pyramid Pooling

To investigate the effect of freezing/tuning layers on TL performance, we examined three levels as the cutoff layers on the TL model. The layers before the identified layer were frozen, whereas those after that were fine-tuned based on the target domain data (Fig. 1). (a) L1: the first layer following the Xception model of the encoder. This was to retain the low-level features learned in the source domain and retrain the high-level features from the target domain. (b) L2: a deep layer following the Atrous Spatial Pyramid Pooling at the end of the encoder. This was to retain the low-level features and most of the high-level features of the encoder in the source domain and retrain the last layer in the encoder. (c) L3: the layer at decoder initiation. This was to retain all the extracted features of the source domain in the encoder and retrain from the start of the decoder.

**Fig. 2:**
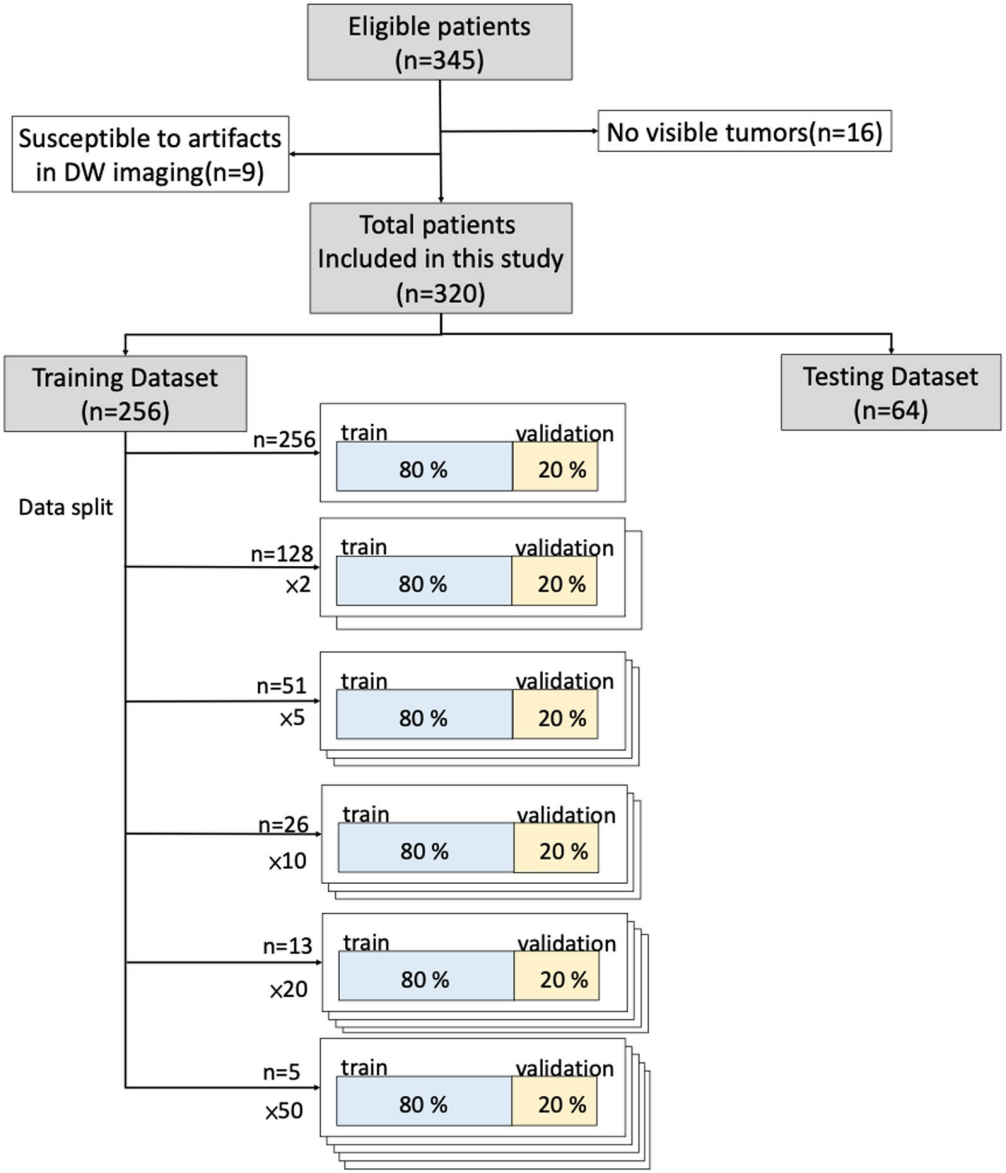
Flowchart of uterine tumor dataset demonstrating data collection and split for various training combinations

To assess the influence of data size on training performance, we examined different training data sizes of uterine dataset through splitting the training samples randomly with 2% (*n* = 5), 5% (*n* = 13), 10% (*n* = 26), 20% (*n* = 51), 50% (*n* = 128), and 100% (*n* = 256) of patients (Fig. 2). The independent dataset comprising patients with uterine cancer (*n* = 64) and cervical cancer (*n* = 25) was used for testing the performance of each group.

### Evaluation of model performances

The accuracy of segmentation was estimated using a dice similarity coefficient (DSC) [19] as follows: ${\text{D}}\left( {{\text{X}},{\text{Y}}} \right) = \frac{{2\left\lfloor {X \cap Y} \right\rfloor }}{\left\lfloor X \right\rfloor + \left\lfloor Y \right\rfloor }$, where *X* and *Y* denote the segmentation of the prediction and ground truth, respectively. The trained models with the highest DSC in each group were selected as the final models for prediction in the testing dataset.

### Extraction of ADC radiomics

To assess the reliability of predicted ROIs by the established models, we examined the radiomics features of ADC values of tumor ROIs extracted by manual and automatic segmentation models. The 14 first-order radiomics features of tumors were calculated using pyradiomics software [20] based on the 3D volumes of ROIs on ADC images.

### Statistics

Statistical analysis was performed using GraphPad Prism software version 8.0 for Mac (GraphPad Software, San Diego, CA, USA). The differences in DSCs in various trained models were assessed using analysis of variance (ANOVA) with Tukey’s post hoc analysis. The stability of the model was assessed through k-fold cross-validations by using ANOVA on DSCs between labeled and predicted ROIs by each trained model. The reliability of radiomics features of tumor ROIs was evaluated using intraclass correlation coefficient (ICC) obtained by manual and automatic segmentation models.

## Results

### Patient characteristics in UT dataset

In total, 320 patients with uterine malignancies were eligible for the final analysis. Table 1 presents the clinical and demographic features of the training (*n* = 256) and testing datasets (*n* = 64). The median patient age was 53 years (range: 25–88 years). The histopathology types comprised endometrial carcinoma (EC, *n* = 309, 96.5%), endometrial stromal sarcoma (ESS, *n* = 5, 1.6%), leiomyosarcoma (LMS, *n* = 4, 1.3%), and carcinosarcoma (malignant mixed müllerian tumor, MMMT, *n* = 2, 0.6%), with tumors either well/moderately differentiated (*n* = 259, 80.9%) or poorly differentiated (*n* = 61, 19.1%). Tumor size ranged from 0.14 to 270 cm^3^ (median, 4.2 cm^3^). No statistically significant differences were found regarding the clinical or demographic characteristics between the training and testing datasets.

**Table 1:** Patient demographics of the uterine dataset

Variable	Training	Testing	*p* value
Patient number (n)	256	64	
Age (year)*	52.14 (11.15)	52.08 (10.77)	0.968
Histology			0.602
Endometroid carcinoma	251 (98.0)	58 (90.6)	
Endometrial stromal sarcoma	2 (0.8)	3 (4.7)	
Leiomyosarcoma	2 (0.8)	2 (3.1)	
Malignant mixed müllerian tumor	1 (0.4)	1 (1.6)	
Differentiation grade			1.000
Well/moderate	207 (80.9)	52 (81.3)	
Poorly	49 (19.1)	12 (18.8)	
T stage			0.811
1	233 (91.0)	57 (89.1)	
2, 3, and 4	23 (9.0)	7 (10.9)	
N stage			0.693
0	238 (93.0)	61 (95.3)	
1	18 (7.0)	3 (4.7)	
M stage			
0	NA	NA	
1	NA	NA	

Data in parentheses are percentages. *Mean (SD)

### Model performance

**Fig. 3:**
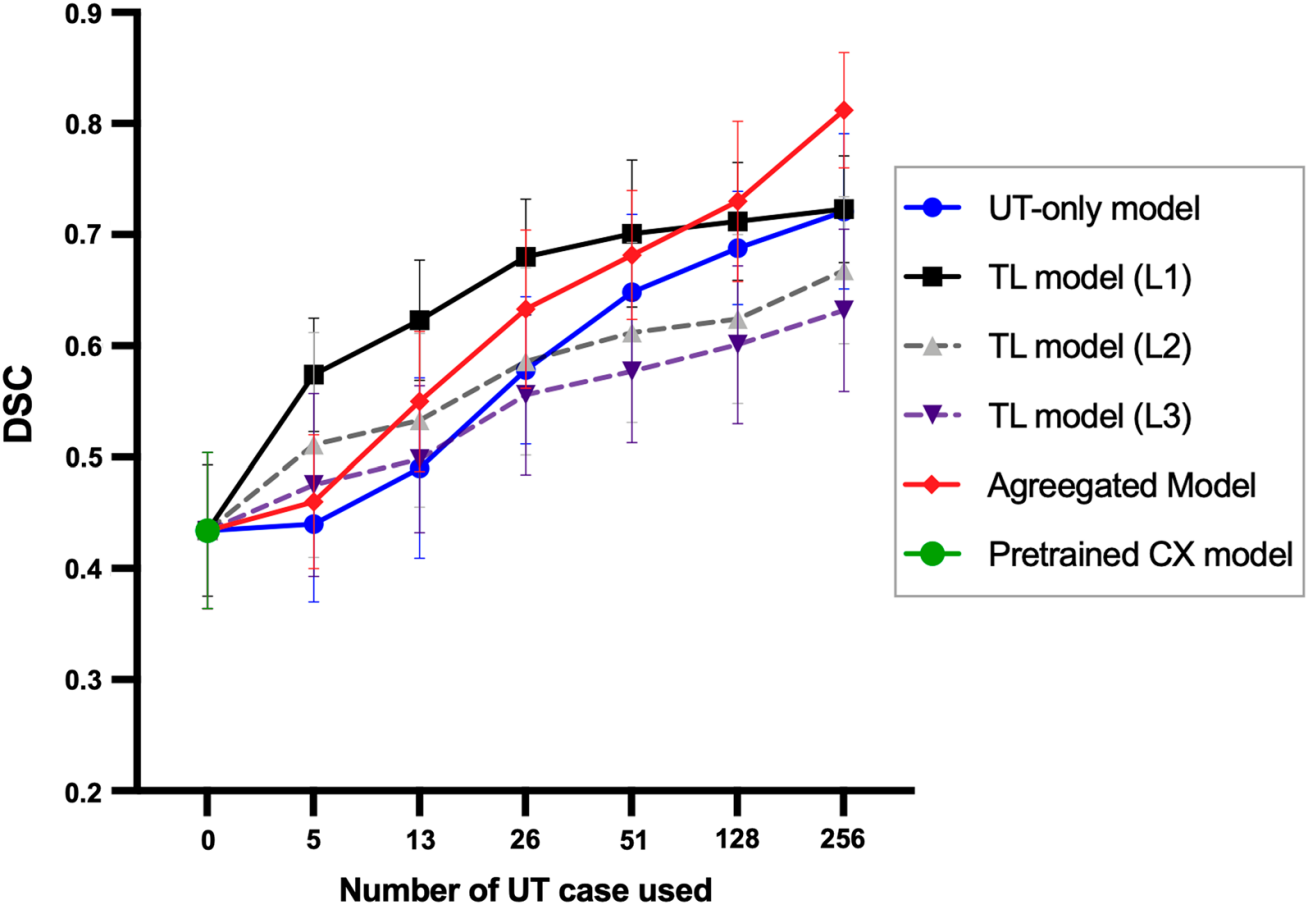
Performances of models in predicting the combined cervical and uterine dataset using various training combinations and sample sizes on the uterine tumors (UT) dataset. The L1, L2 and L3 in TL models indicate the fine-tune levels as indicated in Fig. 1. Data are expressed as means with error bars of standard deviation

**Fig. 4:**
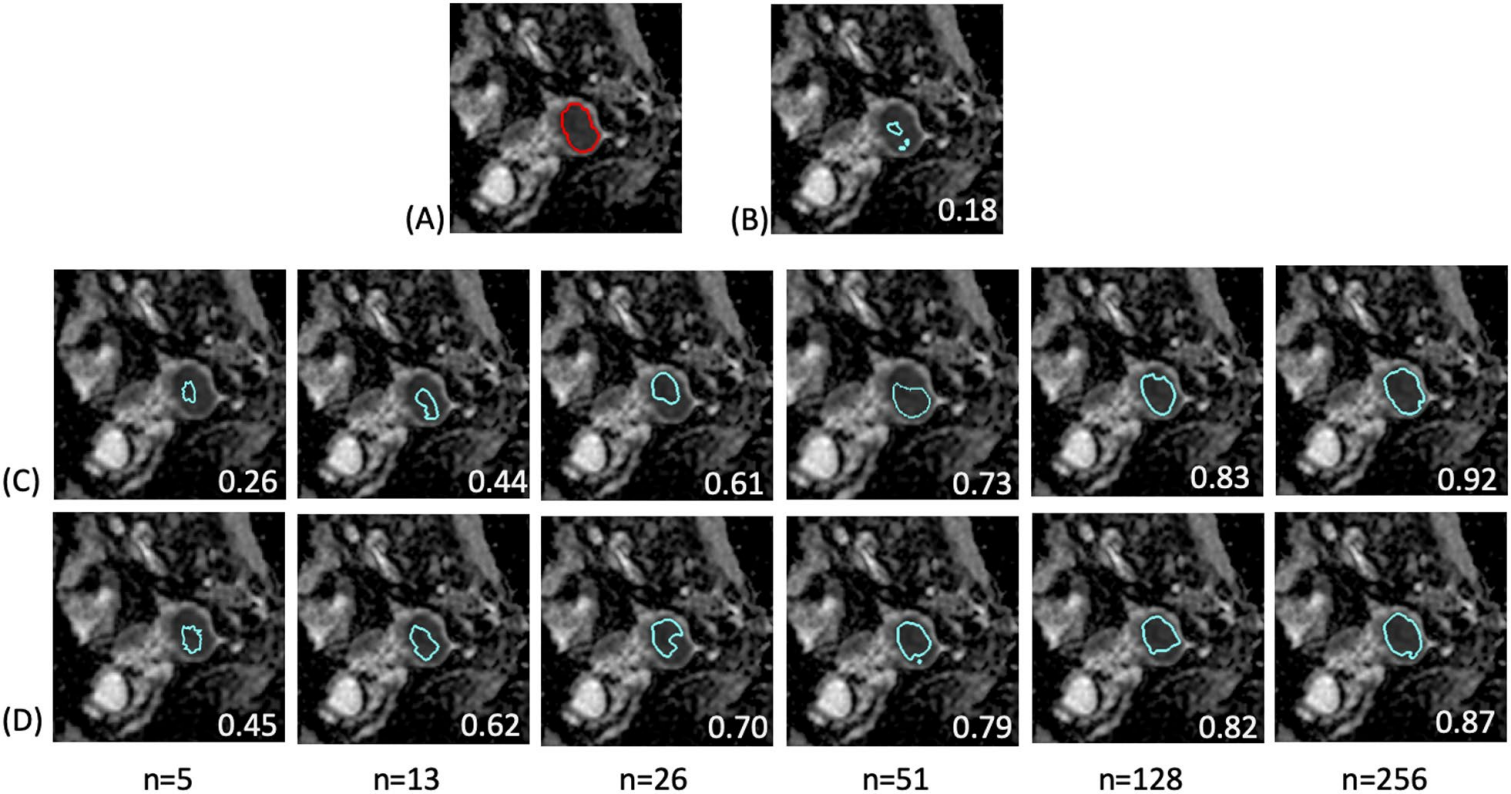
Demonstration of predicted tumor contours in a patient with endometrial cancer using various training combinations and sample sizes on the uterine dataset. **A** The tumor contour was delineated manually (red contour) and overlaid on the ADC image. The blue contours delineate the automatically generated tumor regions by using: **B** pretrained cervical model; **C** uterine-only model; **D** TL model with fine-tuned at L1 level. The numbers in white at the right bottom of each image indicate the DSC of the case. The pretrained cervical model itself generated only a small part of the tumor with DSC = 0.18. The accuracy increased as more uterine data were added for fine-tuning. The TL model outperformed the uterine-only model when the fine-tuned data size was < 128. The uterine-only model exhibited the highest DSC of 0.92 when all patient data were used (*n* = 256)

Figure 3 shows the performance of models in various training combinations and under various sample sizes from the uterine dataset. Initially applying the pretrained cervical model directly to the combined cervical and uterine datasets yielded a DSC of only 0.43 (95% confidence interval [CI], 0.38–0.49). The TL models with the fine-tuning level at L1 exhibited higher DSCs as compared with those at L2 or L3 (*p* < 0.05 for all data size subgroups). The TL model of L1 fine-tune level exhibited the highest DSCs when the used uterine data size was ≤ 51 (DSC = 0.57, 0.62, 0.68, 0.70 for sample sizes 5, 13, 26, and 51, respectively, *p* < 0.001). As more training data were added, the performances of models increased. With the data size of ≥ 128 used, the aggregated model exhibited the highest DSC among all the models (DSC = 0.73 and 0.81 for sample sizes 128 and 256, respectively, *p* < 0.001). Figure 4 demonstrates a patient with endometrial cancer where tumor contours were generated using various training models and sample sizes.

**Fig. 5:**
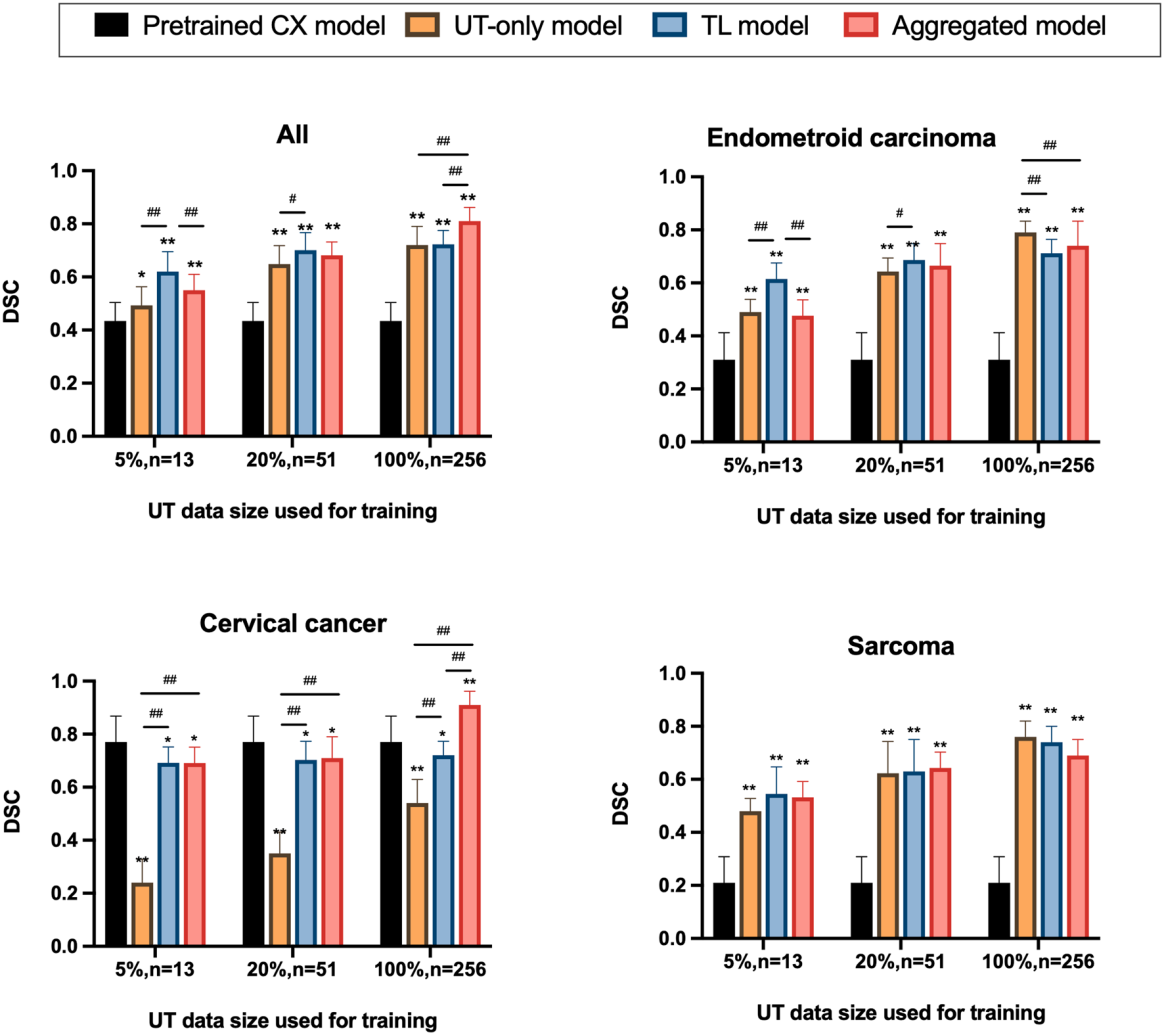
Comparisons of prediction accuracies of various models for subgroup uterine malignancies using various training sample sizes of uterine tumors dataset. The transfer learning (TL) model referred the fine-tuning level of L1. *, significant different compared with the pretrained model; # significant different between the UT-only, TL or aggregated models. * #, *p* < 0.05; **, ##, *p* < 0.001

Subgroup analysis was performed on cervical dataset, uterine dataset, and the combined cervical and uterine datasets, respectively. The prediction accuracies of various models in predicting tumor contours using different training sample sizes are summarized in Fig. 5 and Table 2. Applying the pretrained cervical model directly to uterine dataset yielded a DSC of only 0.31. (95% CI, 0.25–0.34). On testing the uterine cancer cases, the TL model exhibited the highest DSCs when the training size of uterine data was small and medium (DSC = 0.61 and 0.70 for *n* = 13 and 51, respectively) among all models (*p* < 0.001). The UT-only model had the highest DSC when the full dataset was used (*n* = 256, DSC = 0.79, 95% CI, 0.75–0.83, *p* < 0.001). On testing the cervical cancer cases, the TL model achieved similar DSCs as the aggregated model if adding uterine cases of *n* = 13 and *n* = 51 for training (DSC = 0.67 and 0.71, respectively). Surprisingly, the aggregated model drastically improved the DSC in the cervical dataset if adding full uterine cases for training (*n* = 256, DSC = 0.91, 95% CI, 0.87–0.94, *p* < 0.001).

**Table 2:** Prediction accuracies of models for different testing datasets using various training sample sizes

Model	Training data	Test dataset		
		CX (n = 25)	UT (n = 64)	All (n = 89)
UT-only model	UT (n = 13)	0.24 (0.19, 0.28)	0.49 (0.44, 0.54)	0.43 (0.39, 0.48)
	UT (n = 51)	0.35 (0.32, 0.39)	0.65 (0.61, 0.69)	0.62 (0.53, 0.67)
	UT (n = 256)	0.54 (0.49, 0.58)	0.79 (0.75, 0.83)	0.72 (0.67, 0.76)
TL model	UT (n = 13)	0.69 (0.65, 0.73)	0.61 (0.57, 0.65)	0.62 (0.58, 0.66)
	UT (n = 51)	0.71 (0.66, 0.75)	0.70 (0.65, 0.74)	0.70 (0.66, 0.74)
	UT (n = 256)	0.72 (0.68, 0.77)	0.71 (0.67, 0.76)	0.72 (0.68, 0.76)
Aggregated model	CX (n = 144) + UT (n = 13)	0.69 (0.62, 0.74)	0.48 (0.43, 0.53)	0.55 (0.46, 0.65)
	CX (n = 144) + UT (n = 51)	0.71 (0.66, 0.75)	0.66 (0.62, 0.71)	0.68 (0.64, 0.73)
	CX (n = 144) + UT (n = 256)	0.91 (0.87, 0.94)	0.74 (0.71, 0.78)	0.81 (0.76, 0.85)
Pretrained CX model	CX (n = 144)	0.77 (0.73, 0.81)	0.31 (0.25, 0.34)	0.43 (0.35, 0.51)

Data are presented in mean with parentheses for 95% CI

*CX* Cervical dataset, *UT* Uterine dataset

### Reliability of radiomics features

**Fig. 6:**
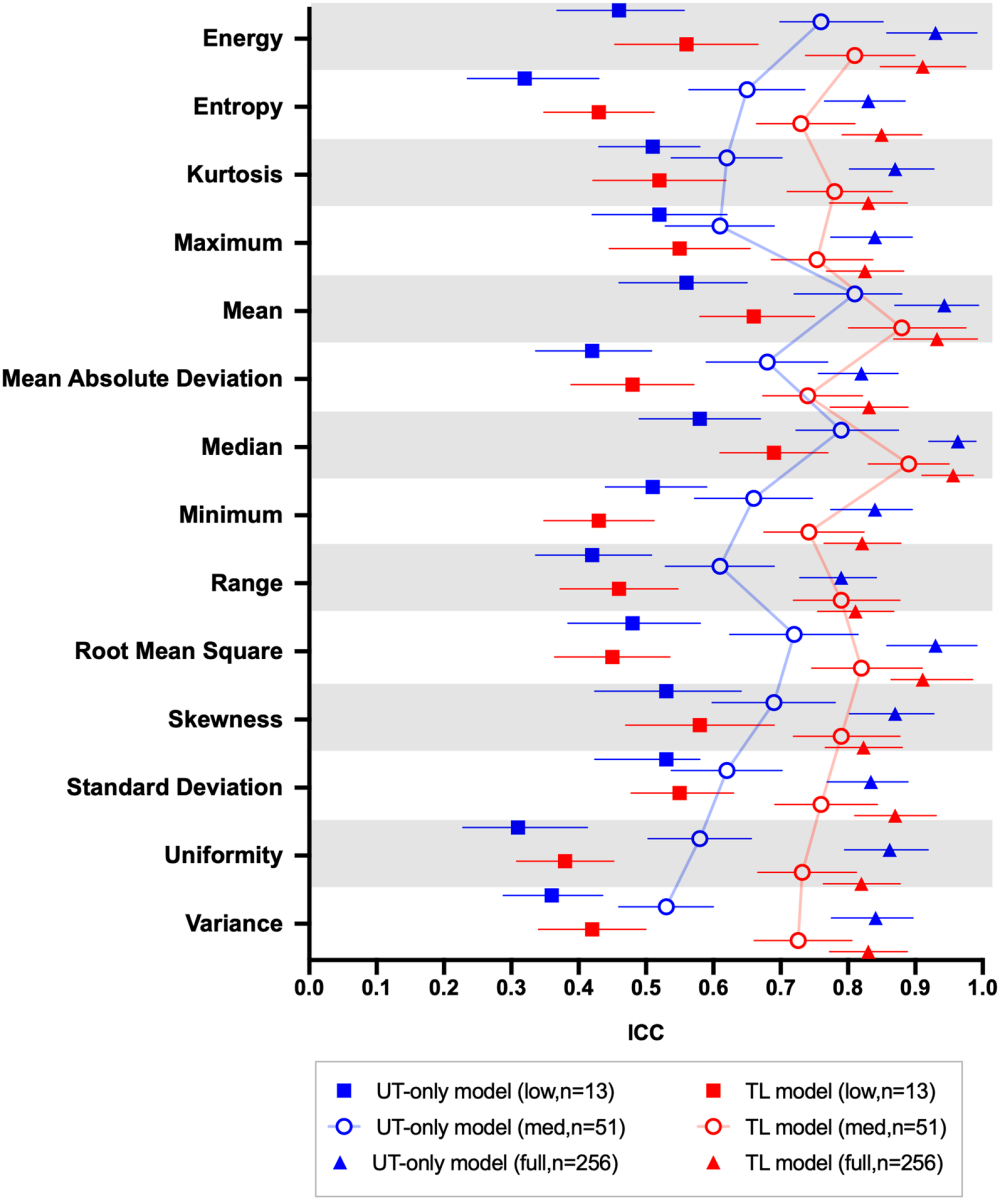
Intraclass correlation coefficient (ICC) values for ADC radiomics features (first-order) obtained by manual and automatic segmentation of uterine (UT)-only and TL models with various sample sizes. Data are presented as median with error bars indicating 95% confidence intervals

Figure 6 shows the ICC values of ADC radiomics features in first-order obtained by manual and automatic segmentation by uterine-only and TL models with various trained sample sizes. Both the models exhibited poor to moderate reliabilities when the training data size was small (*n* = 13) with ICC = 0.32–0.58 for uterine-only model and 0.38–0.69 for TL model. As the training sample size increased to *n* = 51, the TL model exhibited higher ICCs compared with the UT-only model for all parameters (ICC = 0.73–0.89 and 0.53–0.81 for TL and UT-only models, respectively, *p* < 0.001). With the use of full data size, both models exhibited high reliabilities with ICC > 0.8 for all the parameters (ICC = 0.81–0.96 and 0.8–0.96 for TL and UT-only models, respectively).

## Discussion

We exploited the potential of TL through domain adaption for automated tumor segmentation for gynecological cancers on diffusion MRI. Our results showed the effectiveness of the DeepLab V3 + network in tumor segmentation through the adaptation of previously acquired knowledge of cervical cancer to the new domain of uterine malignancy. When the number of training samples was limited in the target dataset, the TL approach outperformed conventional training from scratch with the same size of training data.

TL works under the assumption of a common feature space for data distribution from source and target domains shared. CNN architectures with transferable weights are particularly suited for TL [21, 22]. We hypothesized that cervical cancer and uterine malignancy might have some common features in DW imaging within the scope of gynecology, and the learned weights from the pretrained cervical model might generalize to all cervical and uterine malignancies. Our results showed that model trained from either cervical or uterine tumor failed to predict the contour of the other cancer properly without fine-tuning. Our approach underscored that the cervical cancer and uterine malignancy did share common features that can be learned at low-level layers, whereas the high-level features are specific to the target domain of uterine malignancy. The network can be adopted to the target domain rapidly with only a small sample size to fine-tune the weights.

Our results suggest that with a small sample size, the TL approach outperformed training from scratch for both the segmentation similarity measures as well as the reliability of the extracted radiomics parameters. Kurata et al. [23] demonstrated DSCs of 0.68 and 0.56 in DW imaging and ADC images, respectively, for endometrial cancer by training 180 uterine cancer patients from scratch. Our results showed that, with only 51 patients used, the TL model exhibited higher DSC of 0.70 than the UT-only model with DSC of 0.64. Although the DSC of 0.7 is not satisfactory for the tumor segmentation task, the extracted ADC radiomics is reliable with ICCs of 0.73–0.89. This is nearly comparable with the results by Kurata et al. [23], who reported ICCs of 0.75–0.93 for the first-order features based on T2-weighted image by training 180 patients with endometrial cancer.

The TL approach could be in particular useful for uncommon diseases such as uterine sarcomas demonstrated in the present study. Our finding is consistent with that of Ghafoorian et al. [24], who performed domain adaptation for segmentation on brain white matter among different MRI. They showed that the accuracy of the model using TL outperformed the model trained from scratch with a sample size of < 50. Swati et al. [25] reported that the data size can be reduced to as low as 25% (*n* = 58) by using TL for brain tumor classification on MRI by using the VGG19 network. Our results showed that TL model outperformed all the non-TL models with data size < 128. The potential reason may be that the pretrained model may contain some mutual features for both cervical cancer and uterine malignancy, and these features would dominate the weights of the trained model when the sample size is small.

We also demonstrated that the performance of TL is dependent on fine-tuning layers in the network. In a CNN, the convolutional layers near the input are regarded to extract general features, whereas deeper layers are specific to the target task [26]. Shirokikh et al. [27] reported that fine-tuning the first layers significantly outperforms fine-tuning the last layers in brain segmentation by using U-Net. Our results demonstrated that DeepLab V3 + exhibited higher accuracy compared with U-Net for tumor segmentation in uterine malignancy. We observed that fine-tuning the layers immediately after the Xception portion [28] exhibited the highest performance among the various levels of interest in the network. In addition, fine-tuning at the encoder (L1 and L2 levels) outperformed that at the decoder (L3) of the network. This finding implies that low-level features at the early encoder portion dominate the common features of tumors in cervical cancer and uterine malignancy.

Our study had some limitations. First, we focused on only TL between cervical cancer and uterine malignancy because these two cancers are prevalent in gynecology and the data size available for clinical use is the largest. The value of TL for ovarian cancer is yet to be investigated. Second, we used DeepLab V3 + in this study; innovative networks always exist for semantic segmentation. However, most of the segmentation networks use the encoder–decoder form with various backbones for feature extraction. Nonetheless, the current study provides a proof of concept that fine-tuning from the early part of the encoder is recommended for TL among different cancers. Third, the tumors for radiation planning are segmented on fast spin echo T2-weighted images with higher resolution and signal to noise. Thus, generalizability to other types of datasets needs to also be demonstrated in the future.

In conclusion, our results demonstrated that TL may improve the generalizability of automated tumor segmentation of DWI from a specific cancer type toward multiple types of uterine malignancies especially in limited case numbers. However, if large amounts of annotated data are available, training from scratch using the target dataset appears to be a better option for specific disease.

## Supplementary Information

**Additional file 1:** Comparison of U-Net and DeepLab V3+ architectures for tumor segmentation in cervical cancer.

## Data Availability

The datasets used or analyzed during the current study are available from the corresponding author on reasonable request.

## Abbreviations

ADC: Apparent diffusion coefficient
CNN: Convolutional neural network
CX: Cervical cancer
DSC: Dice similarity coefficient
DW: Diffusion-weighted
EC: Endometroid carcinoma
ESS: Endometrial stromal sarcoma
LMS: Leiomyosarcoma
MRI: Magnetic resonance imaging
ROI: Region of interest
TL: Transfer learning
UT: Uterine tumors
MMMT: Malignant mixed müllerian tumor
