# Supplementary material for: Generalizable transfer learning of automated tumor segmentation from cervical cancers toward a universal model for uterine malignancies in diffusion-weighted MRI
Source: Insights Imaging. 2023 Jan 24;14:14. doi: 10.1186/s13244-022-01356-8 (PMC9871146; doi:10.1186/s13244-022-01356-8)
Supplement: Supplementary file 1 — Additional file 1: Comparison of U-Net and DeepLab V3+ architectures for tumor segmentation in cervical cancer. [file 13244_2022_1356_MOESM1_ESM.pdf]

ELECTRONIC SUPPLEMENTARY MATERIAL

Generalizable Transfer Learning of Automated Tumor Segmentation from Cervical Cancers toward a Universal Model for Uterine Malignancies in Diffusion-Weighted MRI

We explored the performance of U-Net and DeepLab V3+ architectures for tumor segmentation in cervical cancer. A total of 144 patients from the cervical cancer dataset were randomized into the training dataset with varying training data sizes: n = 36 (25%), 72 (50%), and 144 (100%), and another independent 25 patients were used for testing.

The training procedures for U-Net and DeepLab V3+ networks were identical. The regions of interest of tumor contours were delineated by the consensus of two gynecologic radiologist (Y.L.H. and G.L.) using an in-house developed interface in MATLAB (Mathworks, Natick). The labeled ROIs were used as the ground truth for the model training. The diffusion weighted imaging with b-values of 0 and 1000 s/mm^2 and apparent diffusion coefficient (ADC) images were used as three-channel input data for training. The stochastic gradient descent Adam Optimizer method was used to train the networks. The signal intensities of all images were normalized to a mean = 0 and standard deviation = 1. Data augmentation was performed on each training image set, such that six times of image data were generated (20°, −20°, 60°, −60°, and horizontal flip). The learning rate was set to 10^{-5}. The model was trained for 100 epochs for each dataset. The network was trained using Keras 2.1.4 written in Python 3.5.4 and TensorFlow 1.5.0.

The results of the experiments are summarized in Supplementary Table. The DeepLab V3+ model exhibited significantly higher DSCs as compared with U-Net model in all groups ($P < 0.05$).

Supplementary Table

Performances of two network architectures in tumor segmentation for cervical cancer by using various training data sizes. The numbers are DSCs of the testing dataset.

Network model	Data size for training		
	n = 36	n = 72	n = 144
U-Net	0.39 (0.34, 0.44)	0.61 (0.58, 0.65)	0.75 (0.71,0.77)
DeepLab V3+	0.45 (0.41, 0.49)	0.64 (0.60, 0.68)	0.77 (0.73,0.81)

Data are means with 95% CIs in parentheses
